# Spotted Fever Group *Rickettsia* in the Pampa Biome, Brazil, 2015–2016

**DOI:** 10.3201/eid2211.160859

**Published:** 2016-11

**Authors:** Bárbara Weck, Bruno Dall’Agnol, Ugo Souza, Anelise Webster, Barbara Stenzel, Guilherme Klafke, João Ricardo Martins, José Reck

**Affiliations:** Instituto de Pesquisas Veterinárias Desidério Finamor, Eldorado do Sul, Brazil (B. Weck, B. Dall’Agnol, U. Souza, A. Webster, G. Klafke, J.R. Martins, J. Reck);; Centro Estadual de Vigilância em Saúde, Porto Alegre, Brazil (B. Stenzel)

**Keywords:** spotted fever group, SFG, tick, *Amblyomma tigrinum*, Rickettsia, Rickettsiae, R. rickettsii R. rickettsii, Pampa, Rio Grande do Sul, Brazil, bacteria, vector-borne infections

**To the Editor:** Several cases of tickborne rickettsiosis have been reported in South America in recent years (*1*,*2*). In Brazil, 2 spotted fever group (SFG) *Rickettsia* species, *R. rickettsii* and *Rickettsia* sp. strain Atlantic Rainforest, have been identified as causes of human disease. Rio Grande do Sul is the southernmost state of Brazil and the only part of the country located in the Pampa biome. Despite confirmed cases of human spotted fever in that state since 2005, little information is available regarding *Rickettsia* species. We report an eco-epidemiologic investigation of *R. parkeri* in *Amblyomma tigrinum* ticks on dogs from a household (and neighborhood) where a case of human spotted fever was diagnosed.

In 2011, a 44-year-old woman from the municipality of Rosário do Sul in Rio Grande do Sul (Figure) sought medical attention at the municipal health center. On examination, she had a cutaneous eschar, fever, malaise, lymphadenopathy, myalgia, headache, and rash; she reported receiving a tick bite a few days before. The diagnosis of spotted fever was confirmed at the Brazil National Reference Laboratory (Instituto Adolfo Lutz) in São Paulo after paired serologic testing (21-day interval) against *R. rickettsii* (first antibody titration 1:64; second 1:256) because the official diagnosis of human spotted fever in Brazil is based on serologic testing using only the *R. rickettsii* antigen. After doxycycline treatment (2×/d for 7 d), the patient had a complete recovery.

**Figure:**
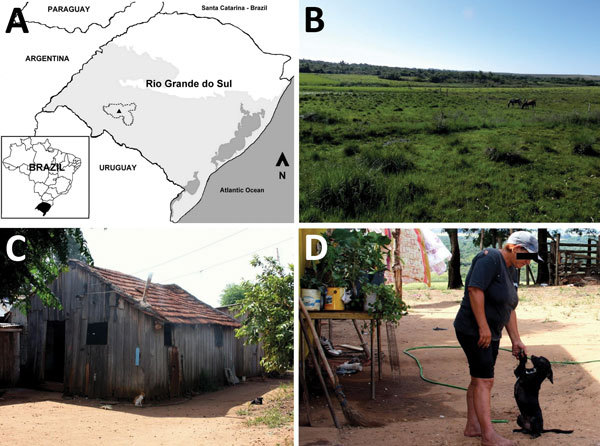
Setting for investigation of human infection with spotted fever group *Rickettsia* in the Pampa biome, Brazil, 2015–2016. A) Rio Grande do Sul state, Brazil, and neighboring countries. Light gray shading indicates the Pampa biome; dark gray shading indicates bodies of water; dotted line indicates Rosário do Sul municipality; black triangle indicates patient’s household. B) Typical view of Pampa vegetation (and area used for hunting by patient). C) Patient’s home. D) The patient and 1 of her dogs in the patient’s backyard.

During September 2015–March 2016, we performed tick collections at the patient’s house, in the surrounding neighborhood (i.e., 7 other homes located within a radius of 1 km), and in the venues used by the patient for hunting. The patient and 11 relatives lived in a small house under extremely poor economic and sanitary conditions. They survived exclusively by government social programs and illegal hunting. The patient usually hunted several wild animals, including capybaras (*Hydrochoerus hydrochaeris*), armadillos (*Dasypus* spp.), the pampas fox (*Lycalopex gymnocercus*), and the crab-eating fox (*Cerdocyon thous*). We collected 251 *Amblyomma dubitatum* ticks from capybaras carcasses (74 adults and 173 nymphs) and from vegetation by dragging/flagging (2 adults and 2 nymphs); 60 *Amblyomma* sp. larvae were obtained by dragging/flagging. We obtained 47 adult *A. tigrinum* ticks and 2 adult *Rhipicephalus sanguineus* ticks from 14 owned free-roaming dogs with permanent access to wild habitats. We obtained ticks from the patient’s 8 dogs and from 6 other dogs from among 3 other households. 

We taxonomically identified the ticks by morphology (*3*), processed whole ticks individually to obtain genomic DNA (*4*), and used PCR amplication of the rickettsial citrate synthase gene (*gltA*) as a screening procedure (*5*). We further tested tick samples that were positive for *Rickettsia* spp. by *gltA* PCR by using a second PCR, which amplified a fragment of the *ompA* gene from SFG *Rickettsia* spp. (*6*). We then tested positive samples a third time by using PCR amplification of a *htrA* gene fragment (*5*,*7*). PCR products of the *ompA* and *htrA* genes were purified and sequenced and then compared with sequences available in GenBank. All samples of *A. dubitatum* ticks were negative. Of the ticks collected from dogs, 13 *A. tigrinum* (28%) and 1 *R. sanguineus* (1/2) were positive in all PCR analyses (*gltA*, *ompA*, *and htrA*); 11 of these ticks were from the patient’s dogs. In all properties where ticks were collected, at least 1 was PCR positive. Thus, we detected *R. parkeri* in half (4/8) of investigated households. 

All the sequences generated for the *ompA* and *htrA* genes showed 100% identity to sequences from the *Rickettsia parkeri* strain Portsmouth (GenBank accession no. CP003341.1). We deposited into GenBank the sequences of the *ompA* gene (KX196265) and *htrA* gene (KX196266) from samples analyzed in this study. The *ompA* sequence we obtained for *R. parkeri* showed 98% identity with *Rickettsia* sp. strain Atlantic Rainforest (GenBank accession no. GQ855237.1). 

Although *Rickettsia* sp. strain Atlantic Rainforest had previously been considered the only SFG *Rickettsia* in southern Brazil, we demonstrate here the presence of *R. parkeri* in Rio Grande do Sul in the Pampa biome. We detected *R. parkeri* infection in *A. tigrinum* ticks collected at the probable site of infection (the patient’s home) of a confirmed case of human spotted fever. Considering the *A. tigrinum* tick abundance in southern Brazil and its remarkable ability to parasitize domestic and wild animals (*8*), in addition to the high *R. parkeri* infection rate observed (28%), further epidemiologic studies are needed to address the role of *A. tigrinum* ticks as vector of spotted fever in the Pampa biome. Finally, our results show that, in addition to *R. rickettsii* and *Rickettsia* sp. strain Atlantic Rainforest, *R. parkeri* occurs and might be associated with cases of spotted fever in Brazil. Additional surveys are needed to assess the infection prevalence of *R. parkeri* in *A. tigrinum* ticks in other areas of Pampa and in other regions of Brazil. 
